# Arterial Stiffness—What Do We Know about It and What Do We Expect from This Biomarker?

**DOI:** 10.3390/jcm11164843

**Published:** 2022-08-18

**Authors:** Željko Reiner

**Affiliations:** Department of Internal Medicine, University Hospital Center Zagreb, 10000 Zagreb, Croatia; zreiner@kbc-zagreb.hr

It is well known that arterial stiffening is one of the earliest detectable signs of structural and functional alterations of the arterial wall. It occurs as a result of aging but also occurs prematurely, caused by atherosclerosis as a result of different cardiovascular risk factors, e.g., hypertension, diabetes, smoking, obesity or chronic kidney disease, because of which media of the arterial wall undergoes structural and functional changes, resulting in reduced distensibility. The loss of elasticity and increased rigidity results in increased velocity of pulse waves since they travel faster in stiff arteries. Many studies have reported different physiological and pathophysiological conditions associated with increased arterial stiffness [1]. For instance, it has been shown that arterial stiffness is increased not only in atherosclerotic cardiovascular disease but also in systemic sclerosis, in autoimmune diseases characterized by endothelial dysfunction and fibrosis of skin and visceral organs [2], in inflammatory bowel disease which is accompanied with endothelial dysfunction [3] as well as in subclinical and overt hypothyroidism, hyperthyroidism and in primary hyperparathyroidism. It seems that levothyroxine replacement therapy causes an improvement in arterial stiffness in patients with hypothyroidism and that arterial stiffening is reversed by parathyroidectomy in primary hyperparathyroidism [4]. It has to be stressed that thyroid and parathyroid diseases are characterized by an increased cardiovascular risk.

Some of the studies on arterial stiffness have been published most recently shedding a new light on this topic. Literature data suggested a correlation between serum magnesium levels in the upper part of the distribution and lower arterial stiffness parameters [5]. However, most recent findings suggest that ionized magnesium should be used as a more specific marker of the magnesium status indicating its association with arterial stiffness [6].

A very interesting recently published systematic review suggested an association between gut microbiota composition and arterial stiffness, with two patterns in most animal and human studies—a direct correlation between arterial stiffness and abundances of bacteria associated with altered gut permeability and inflammation; an inverse relationship between arterial stiffness, microbiota diversity, and abundances of bacteria associated with most fit microbiota composition. However, interventional studies showed a stable association between microbiota modification and arterial stiffness only in animals but no human interventional trial was able to demonstrate this relationship [7].

It has been mentioned already that obesity is also associated with an increased arterial stiffening. Therefore, it is not a surprise that a recent meta-analysis has shown that the arterial stiffness is improved after bariatric surgery in extremely obese subjects [8]. A recent study showed that arterial stiffening precedes arterial wall thickening and that it begins at the overweight stage already in childhood or early adolescence [9]. This is in accordance with epidemiological studies which have reported that childhood obesity is associated with adverse vascular alterations in adulthood [10]. When discussing childhood obesity, which is associated with consumption of different unhealthy types of food and drinks, it has to be mentioned that children and adolescents are the main consumers of energy drinks. The results of a recent study showed that acute energy drinks consumption might be associated with increased arterial stiffness in healthy children and teenagers. Therefore, minors, especially those with increased risk for cardiovascular diseases, should be discouraged from energy drink consumption [11].

Particularly interesting question is whether arterial stiffness, as an established independent predictor of cardiovascular risk, is increased in patients with familial hypercholesterolemia (FH). FH is, because of life-long exposure to elevated serum levels of LDL-cholesterol, the most important risk factor for accelerated and premature atherosclerotic cardiovascular disease. A number of studies demonstrated a significant positive relationship between artery stiffness and total cholesterol and/or LDL-cholesterol and they particularly showed an increased arterial stiffness in patients with FH [12]. A meta-analysis showed that LDL-cholesterol-lowering therapy with statins causes a significant reduction in arterial stiffness which is independent of LDL-cholesterol changes [13]. However, the results of a recent meta-analysis suggest that patients with FH do not have significantly altered arterial stiffness when compared with normocholesterolemic subjects [14]. Therefore, the question whether patients with FH do have an increased arterial stiffness or not still remains unanswered. The same dilemma exists when children are in question. Some studies have found increased arterial stiffness in children with FH and some have not. A most recent position paper focusing on risk assessment and clinical management of children and adolescents with heterozygous FH still recommended that depending upon the availability of arterial stiffness measurement and staff experience, it would be clinically meaningful to perform arterial stiffness estimation in all children with FH and to evaluate their changes over time [15].

Nevertheless, increased arterial stiffness is associated not only with risk factors for atherosclerosis. It has been shown recently that arterial stiffness might be a strong prognostic parameter in heart failure patients discharged after an acute heart failure decompensation [16].

As a conclusion, it could be stated that arterial stiffness is more and more in the focus of interest of different studies trying to find whether this might be another reliable biomarker for early detection of increased cardiovascular risk and the accumulated data seem to be very much in favor of a positive answer.
